# Museomics and morphological analyses of historical and contemporary peninsular Italian wolf (*Canis lupus italicus*) samples

**DOI:** 10.1038/s41598-024-84319-x

**Published:** 2025-02-04

**Authors:** Elena Fabbri, Antonia Vecchiotti, Federica Mattucci, Edoardo Velli, Vilde Arntzen Engdal, Nicola Baccetti, Adriano De Faveri, Pavel Hulva, Barbora Černá Bolfíková, Urmas Saarma, Elisabetta Cilli, Romolo Caniglia

**Affiliations:** 1 Unit for Conservation Genetics (BIO-CGE), Italian Institute for Environmental Protection and Research (ISPRA), Via Cà Fornacetta 9, Bologna, 40064 Ozzano dell’Emilia Italy; 2 Biodiversity Management and Monitoring Office, Majella National Park, via Badia 28, Sulmona, 67039 L’Aquila Italy; 3 Department of Preclinical Sciences and Pathology, Faculty of Veterinary Medicine, Norwegian University of Life Sciences, Ås, Norway; 4 Zoological Museum, Italian Institute for Environmental Protection and Research (ISPRA), Via Cà Fornacetta 9, Bologna, 40064 Ozzano dell’Emilia Italy; 5 Department of Zoology, Faculty of Science, Charles University, Viničná 7, Prague 2, 128 43 Czech Republic; 6 Faculty of Tropical AgriSciences, Czech University of Life Sciences, Kamýcká 129, Prague, 16500 Czech Republic; 7 Department of Zoology, Institute of Ecology and Earth Sciences, University of Tartu, J. Liivi 2, Tartu, 50409 Estonia; 8 Department of Cultural Heritage, Alma Mater Studiorum-University of Bologna, via degli Ariani 1, Ravenna, 48121 Italy

**Keywords:** *Canis lupus italicus*, Conservation management, Genetic variability patterns, Historical biological samples, Apennine Italian wolves, Museomics, Museum collections, Population genetics, Multilocus genetic profiles, Population genetics, Evolutionary biology, Genetic hybridization, Conservation biology, Population dynamics

## Abstract

After centuries of decline and protracted bottlenecks, the peninsular Italian wolf population has naturally recovered. However, an exhaustive comprehension of the effects of such a conservation success is still limited by the reduced availability of historical data. Therefore, in this study, we morphologically and genetically analyzed historical and contemporary wolf samples, also exploiting the optimization of an innovative bone DNA extraction method, to describe the morphological variability of the subspecies and its genetic diversity during the last 30 years. We obtained high amplification and genotyping success rates for tissue, blood and also petrous bone DNA samples. Multivariate, clustering and variability analyses confirmed that the Apennine wolf population is genetically and morphologically well-distinguishable from both European wolves and dogs, with no natural immigration from other populations, while its genetic variability has remained low across the last three decades, without significant changes between historical and contemporary specimens. This study highlights the scientific value of well-maintained museum collections, demonstrates that petrous bones represent reliable DNA sources, and emphasizes the need to genetically long-term monitor the dynamics of peculiar wolf populations to ensure appropriate conservation management actions.

**Supplementary Information:**

The online version contains supplementary material available at 10.1038/s41598-024-84319-x.

## Introduction

Despite their key role in regulating ecosystem equilibria, wolves (*Canis lupus*) have experienced centuries of worldwide severe demographic declines^1,2^, mainly due to habitat loss and human persecution, which led some populations to the verge of local extinction^3,4^. However, thanks to legal protection, habitat restoration and recovery of natural prey, wolves are numerically increasing and geographically re-expanding across their historical ranges, both in remote and rural semi-urbanized areas^1,5^. These rapid demographic recovery trends have prompted a number of ecological and molecular studies. These studies aimed to address management conservation issues due to conflicts with human activities and anthropogenic threats, such as wolf *x* dog hybridization, and better investigate the biology, ecology and population dynamics of this flagship species, that despite all is still poorly known^6,7^.

Nevertheless, most of these studies did not include historical data and principally focused on contemporary wolf populations to mainly describe their current patterns of morphological variability and genetic structure, thus showing limited resolutions about (a) the historical causes that determined them^8^ and (b) the evolutionary scenarios that such populations experienced during demographic contractions and re-expansion processes^9^, which were mostly deduced only from recent patterns^10^.

Historical collections from natural history museums (NHM) can theoretically help to fill such gaps of information, especially for populations that experienced recurrent bottlenecks, expansions, replacements or introgression, through the morphological observation and molecular analysis of biological materials like skins, skulls, bones, claws and teeth collected from animals living in the past or belonging to extinct taxa^11,12^. However, NHM collections can rarely provide multiple individuals from the same population^13^. Additionally, museum samples are often precious and fragile, and usually contain fragmented and low-quality DNA due to natural post-mortem processes and preservation methods^14^. Fortunately, recent methodological improvements and analytical advances can produce reliable genetic data even from such degraded samples^15,16^, exploiting the possibility to extract well-preserved ancient endogenous DNA from particularly dense mammal bones of the temporal region such as the petrous bone (*pars petrosa*)^17^.

Consequently, ancient and historical museum DNA has been successfully applied in several studies providing useful management conservation insights for threatened or critically endangered species^18–21^.

In this study we applied a multidisciplinary approach, based on morphological and genetic analyses performed on both historical and contemporary samples, also exploiting the optimization of an innovative bone DNA extraction method. Such approach was utilized to describe the morphological variability of the peninsular Italian wolf population and its genetic diversity during the last 30 years. Such a population symbolizes an unquestionable example of a recent conservation success. After being close to extinction in the 1970s^22^, with only about 100 individuals surviving in the central-southern Apennines^22^, in the 1980s it started a natural re-colonization process along the Apennines, mainly thanks to the ecological plasticity of the species, legal protection and prey availability. This process led the population to reach the western Alps in the 1990s^23^ and the central-eastern Alps in the 2010s^8,24^, where occasional gene flow from neighbouring Dinaric and/or Carpathian populations^25^ occurred, contributing to the genetic composition of current central European wolf populations^26^.

Additionally, the peninsular Italian wolf population, currently numbering at least 3000 individuals^27^, also represents a fascinating taxonomic uniqueness^28^, since protracted geographic isolation in the glacial refugium south of the Alps and recurrent demographic bottlenecks made it morphologically, genetically and genomically differentiated from any other worldwide wolf population^29–31^, to be recently confirmed as a distinct subspecies (*C. l. italicus* Altobello, 1921^32^).

In recent years, many studies have been carried out to better understand the evolutionary potential, ecological role, pack dynamics and ongoing threats to the long-term conservation of the species, such as wolf-dog hybridization^33^ and anthropogenic mortality causes^34^. However, only a single study has systematically described the morphological peculiarities of the subspecies^35^, and a few studies have investigated its past genetic variability patterns, but they were mainly based only on mitochondrial DNA (mtDNA) analysis of a very limited number of samples^36–39^. In this study, we exploited the availability of (1) a well-preserved and annotated museum historical collection (ISPRA zoological collection, Ozzano Emilia, Italy), consisting of dozens of Apennine wolf skins and skulls belonging to animals that lived during the last 30 years (Table 1), (2) a large database, including more than 300 historical and contemporary wolf and dog multilocus genotypes obtained from DNA extracted from found-dead and injured animals collected throughout the entire peninsular Italian wolf range distribution during the last three decades (ISPRA *Canis* database^28,40^), and (3) a reliable canid multi-marker panel well-discriminating wolves, dogs and their first hybrid generations^33^. We applied these tools aiming to: (1) morphologically describe the peninsular Italian wolf population; (2) investigate potential significant changes of its genetic variability through time, from the 1990s until nowadays, focusing on samples collected in a sector of its historical core distribution area, where the species never disappeared^22^; (3) evaluate the multilocus genotyping success rate of historical wolf DNA obtained through a recently emerging ancient DNA extraction technique^41^, widely applied in paleogenomic studies, but never tested on wild canid museum samples^42^.

**Table 1:** Information about sample types used in the study.

Sample type	Sample size	Collection years	Sex	Genetic analyses				Morphometric analyses		
				Tissue samples	Blood samples	Petrous bones	Petrous bones and tissues	Skull	Skin	Body measures
Historical wolves (HW)	57	1993–2000	M	28		2	2	21 (12 Adults)	10	8
			F	19	1	4		17 (8 Adults)	9	6
			ND			1		1	1	
Contemporary wolves (CW)	56	2020–2024	M	25	3					17
			F	24	4					8

For each biological category (tissue, blood, or petrous bone samples) and type of analyses (genetic or morphological), number of samples, years of collection and sex (M = male; F = female; ND = not detected) are reported.

## Materials and methods

### Data availability

The majority of the data generated and analyzed during the current study are presented within the article or in Supplementary information files. The raw data are available from the corresponding author on reasonable request.

### Ethical statements

No ethics permit was required for this study, and no animal research ethics committee prospectively was needed to approve this research or grant a formal waiver of ethics approval since the collection of wolf samples involved dead animals. Fieldwork procedures were specifically approved by ISPRA as a part of national wolf monitoring multi-year activities^27^.

Dog blood samples were collected by veterinarians during health examinations with a not-written (verbal) consent of their owners (students/National Park volunteers/or specialized technician personnel of the Italian Forestry Authority (CFS)), since they were interested in wolf conservation studies and monitoring projects in Italy. Moreover, there is not a relevant local law/legislation that exempts our study from this requirement.

Additionally, no anesthesia, euthanasia, or any kind of animal sacrifice was applied for this study and all blood samples were obtained aiming at minimizing the animal suffering.

### Sample collection and DNA extraction

**Fig. 1:**
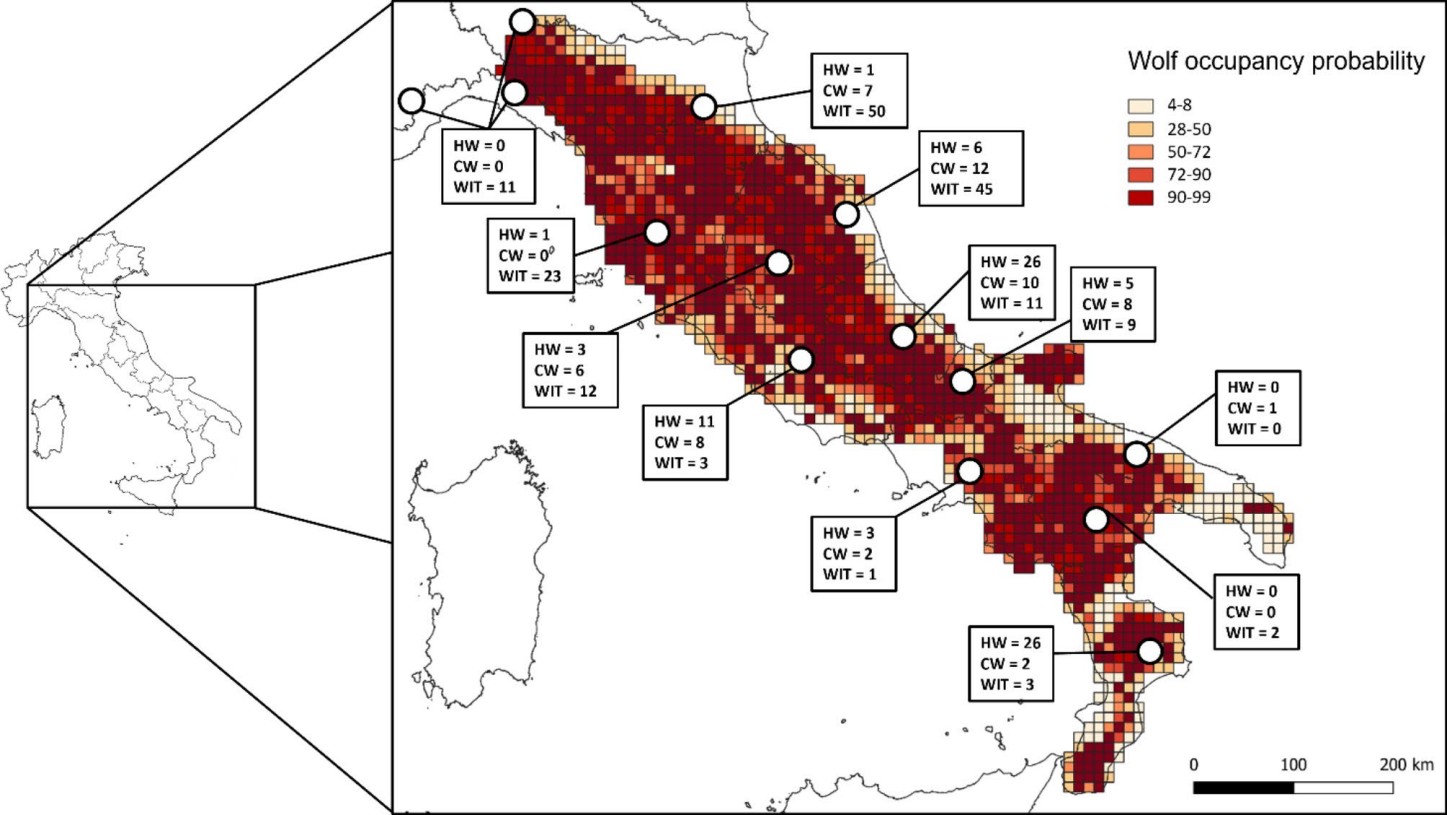
Map visualizing the geographical distribution and sampling locations of the Italian reference (WIT), peninsular historical (HW) and contemporary (CW) wolf samples analyzed in this study. Wolf distribution and occupancy probability estimates are derived, according to the policy of the Widely publisher group about Creative Common CC BY license, from Gervasi et al.^27^, and using a 10 × 10 km grid adopted at the European level for the Habitats Directive 92/43/EEC reporting (https://www.eea.europa.eu/data-and-maps/data/eea-reference-grids-2).

We molecularly analyzed 113 presumed wolf biological samples (Table 1 and Table S1), opportunistically collected from individuals found dead or injured across the central-southern Apennine range of the species (Fig. 1), selected from the ISPRA *Canis* biobank^33^. Biological materials included 57 samples (24 females, 32 males and 1 not sex determined) collected from 1993 to 2000 (roughly corresponding to about two wolf generations), namely during the beginning of the population re-expansion phase, occurred after the last population bottleneck^22^, (hereafter referred to as historical wolf samples, HW). Additionally, biological materials included 56 samples (28 females and 28 males) collected from 2020 to 2024 (corresponding to a single actual wolf generation), namely the period when the species has presumably saturated all the ecologically suitable mountain and rural areas^27^ (hereafter referred to as contemporary wolf samples, CW). For 47 HW and 49 CW, a fragment of about 1 cm^3^ of muscular tissue was cut and stored in 50 ml of ethanol 95% at − 20 °C, for 1 HW and 7 CW, 1 ml of fresh blood was collected and preserved in EDTA solution at − 20 °C. For 7 HW samples, the entire petrous bone was collected and stored at 4 °C. For 2 HW individuals, DNA samples were independently derived from both tissue and petrous bone sources to check for the real quality of skeletal DNA and its applicability in genotyping procedures (Table 1 and Table S1).

Furthermore, for 19 HW (8 females, 11 males) the entire skull and for other 20 HW (9 females, 11 males) both the skull and the skin were available in the ISPRA zoological collection, Ozzano dell’Emilia, Italy, and were thus used to perform descriptive craniometric and morphometric analyses (Table 1 and Table S1). For comparative purposes, we also morphologically analyzed 8 dog skulls (1 female, 4 males and 3 not sex determined) available at the ISPRA Zoology Museum, belonging to wolf-sized breeds. No skull nor skin data were available for the 56 CW (Table 1 and Table S1).

Muscular and blood DNA was extracted using the Qiagen DNeasy Blood & Tissue Kit (Qiagen, USA), following the manufacturer’s instructions.

DNA content from all the 9 petrous bones was extracted using an innovative procedure based on a silica protocol^43,44^, specifically designed for ancient samples, performed in a room solely dedicated to the manipulation of skeletal elements and degraded DNA, equipped with positive air pressure with HEPA filters and laminar flow cabinets, following ancient DNA guidelines^11^. The complete list of the pursued criteria for ancient DNA studies has been extensively described elsewhere^45^.

Double-stranded DNA concentrations from petrous bone samples were quantified using the Qubit^®^ dsDNA HS (High Sensitivity) Assay Kit (Invitrogen™Life Technologies - Carlsbad, CA, USA).

### Molecular analyses

#### Multilocus genotype reconstruction

DNA samples were genotyped using a panel of 39 canine unlinked autosomal microsatellites (STR), that, thanks to their high polymorphism, have been already successfully applied to perform individual identifications^40^, clarify the genetic structure of the European wolf populations^28^, solve forensic cases^46,47^ and reliably discriminate between wolves, dogs and their first three generation hybrids through Bayesian assignment procedures^33^.

Each individual multilocus profile was completed by the amplification of the *Amelogenine* gene, to molecularly determine its sex, and of the K*-*locus marker on the CBD103 gene, which is associated with the black coat colour in canids^48,49^. Finally, 4 Y-chromosome STRs (MS34A, MS34B, MS41A and MS41B^50^, and a 498-bp long fragment of the mitochondrial DNA control region (mtDNA CR^51^) were amplified to reconstruct paternal and maternal haplotypes and characterize uniparental lineages. DNA from carcasses opportunistically collected, blood samples from injured animals and museum specimens was amplified at autosomal loci and Y-linked STRs following a multi-tube approach^52^. The multiple amplifications per sample per locus were performed in seven multiplexed reactions using the QIAGEN Multiplex PCR kit (Qiagen Inc., Hilden, Germany) in a total volume of 10 µL, containing 1 µL of DNA, 5 µL of MasterMix, 1 µL of Q-solution, 0.10–0.30 µl of primers and RNAse-free water up to the final volume, using the following thermal profile: 94 °C for 15 min, 94 °C for 30 s, 57 °C for 90 s, 72 °C for 60 s (40 cycles for petrous bones, and 35 cycles for muscle and blood samples), followed by a final extension step of 72 °C for 10 min.

Mitochondrial sequences were amplified in a total volume of 10 µL, containing 1 µL of DNA solution, 0.3 pmol of the primers WDLOOP and H519^53^, using the following thermal profile: 94 °C for 2 min, 94 °C for 15 s, 55 °C for 15 s, 72 °C for 30 s (40 cycles), followed by a final extension of 72 °C for 5 min. PCR products were purified using exonuclease/shrimp alkaline phosphatase (Exo-Sap; Amersham, Freiburg, Germany) and sequenced in both directions using the Applied Biosystems Big Dye Terminator kit (Applied Biosystems, Foster City, California) with the following steps: 96 °C for 10 s, 55 °C for 5 s, and 60 °C for 4 min of final extension (25 cycles).

PCR products were analyzed in an ABI 3130XL automated sequencer. The allele sizes of the STR loci were estimated using the ABI ROX-350 and LIZ500 size standards and the ABI software Genemapper v.4.0. We ran Genemapper following all the recommendations of the Process Quality Value Tests for basic troubleshooting about stutters, quality, weight and width of allele peaks and applying Bin Alleles defined using only good-quality canid DNA samples. For further details on PCR conditions and thermal profiles see Caniglia et al. (2013)^49^. Sequences were visually edited using the ABI software SeqScape v.2.5 and aligned with BioEdit^54^. Identical haplotypes were matched using DnaSP v.5.0^55^ and compared with sequences available from GenBank using Blast^56^.

Extraction of DNA and set up of amplification of museum and muscular/blood samples were carried out in separate rooms reserved to low-template DNA samples, adding a blank control (no biological material) during DNA extraction, and a blank control (no DNA) during DNA amplification. PCR runs and post-PCR laboratory procedures were carried out in a dedicated laboratory, physically separated from the pre-PCR area. Moreover, due to the intrinsic degraded condition of historical/ancient DNA, multiple extractions, independent amplifications and further sequencing were performed to improve the detection of the damaged sites.

#### Amplification success, error rates and reliability analysis

Consensus genotypes were reconstructed from the two replicates per locus foreseen by the multiple-tube approach using Gimlet v.1.3.3^57^, accepting heterozygotes only if both alleles were seen in the two replicates, and homozygotes only if a single allele was seen in the two replicates. Gimlet was also used to calculate PCR success rate (PCR+: number of successful PCRs divided by the total number of PCR runs across samples), allelic drop-out (ADO: number of times a heterozygous genotype failed to amplify at a given locus) and false alleles (FA: number of times one or more false alleles were produced at a locus over the total number of successful amplifications^58^).

#### Genetic population structure and admixture analysis

The potential non-Italian origin of some HW, the possible presence of HW or CW showing signals of admixture with the domestic dogs and hypothetical patterns of differentiation among historical and contemporary wolves were evaluated using two different methodological approaches: (1) a principal component analysis (PCA) in R 4.3.2 with the Adegenet package 2.1.10^59^, and (2) a Bayesian clustering procedure, implemented in the program Structure v.2.3.4^60^, which estimates, comparing to genetic profiles of reference populations, the admixture proportion of each individual genotype, independently of any prior non-genetic information.

We selected from the ISPRA *Canis* multilocus genotype database, as reference populations, 89 wolf-sized free-ranging dogs from rural areas of central Italy, 175 Italian (including samples from both peninsular and Alpine wolf populations collected from 1987 to 2019), 92 Dinaric, 19 Iberian, 23 Carpathian, 38 Baltic and 26 Balkan wolves^28,33^. All the selected wolves showed neither morphologically nor genetically detectable signs of hybridization^28^. Multivariate and Bayesian clustering analyses were first performed using all the reference dog and wolf populations and successively focusing only on Italian canids. The Principal Component Analysis was run using the “*dudi.pca*” function and graphically visualized with the “*s.class*” function. The eigenvalues of the analysis, indicating the amount of variance represented by each principal component (PC), were further plotted using the “add.scatter.eig” function. When considering all the European wolf populations, Structure was run for *K* values ranging from 1 to 10, whereas when considering the Italian context we selected *K* = 2 (corresponding to the optimal number of clusters separating dogs and wolves^33,61^), in both cases with four independent replicates per *K* and using 500,000 Markov chain Monte Carlo (MCMC) iterations, after a burn-in of 50,000 iterations, assuming no prior information (option “*usepopinfo*” not activated), and choosing the “*Admixture*” (each individual can have ancestry in multiple parental populations) and the “*Independent Allele Frequency*” models.

Clumpak^62^ was used to (a) identify the highest rate of increase in the posterior probability LnP(*K*) between consecutive *K* values corresponding to the optimal *K*-value, (b) to assess the average (*Q*_i_) and individual (*q*_i_) proportions of membership in each cluster from the four MCMC replicates, and to graphically display the results.

When considering all the European wolf populations, HW and CW individual genotypes were assigned to the reference Italian wolf, European wolf or dog clusters (see Results) at threshold *q*_i_ > 0.900^28^, whereas when considering the Italian context, HW and CW individual genotypes were assigned to the reference Italian wolf or dog clusters at *q*_i_ ≥ 0.995, as introgressed individuals at 0.955 ≤ *q*_i_ < 0.995, or as recent hybrids at *q*_i_ < 0.955, following criteria described in Caniglia et al. (2020)^33^. Assignments were integrated with the information derived from the uniparental (mtDNA, 4 Y-linked STRs) and coding (K-locus) markers, which were used to confirm the taxon identification or, in case of admixed individuals, to provide the directionality of the hybridization or introgression^40,63^.

#### Genetic variability analysis

The proportions of polymorphic (PL) and monomorphic (ML) loci per group (HW and CW), numbers of observed (N_A_) and effective (N_E_) alleles, observed and expected heterozygosity (H_O_ and H_E_), numbers of rare (0.001 < allele frequency < 0.05; N_R_) and private (exclusively of a population; N_P_) alleles, and analysis of molecular variance (AMOVA) were computed using GenAlEx 6.502. Allelic richness (A_R_), which corrects the observed number of alleles for differences in sample sizes, was computed with FSTAT 2.9.3.2. Values of the inbreeding coefficient Wright’s *F*_IS_ and departures from Hardy-Weinberg equilibrium (HWE) were computed in Genetix v.4.05^64^ using 10,000 random permutations to assess significance levels. Finally, the significance of the differences of the genetic variability indexes among all the analyzed wolf populations was assessed using an analysis of variance (ANOVA) performed in PAST v.3.26 software^65^.

### Morphometric analyses

#### Skull morphometry

**Fig. 2:**
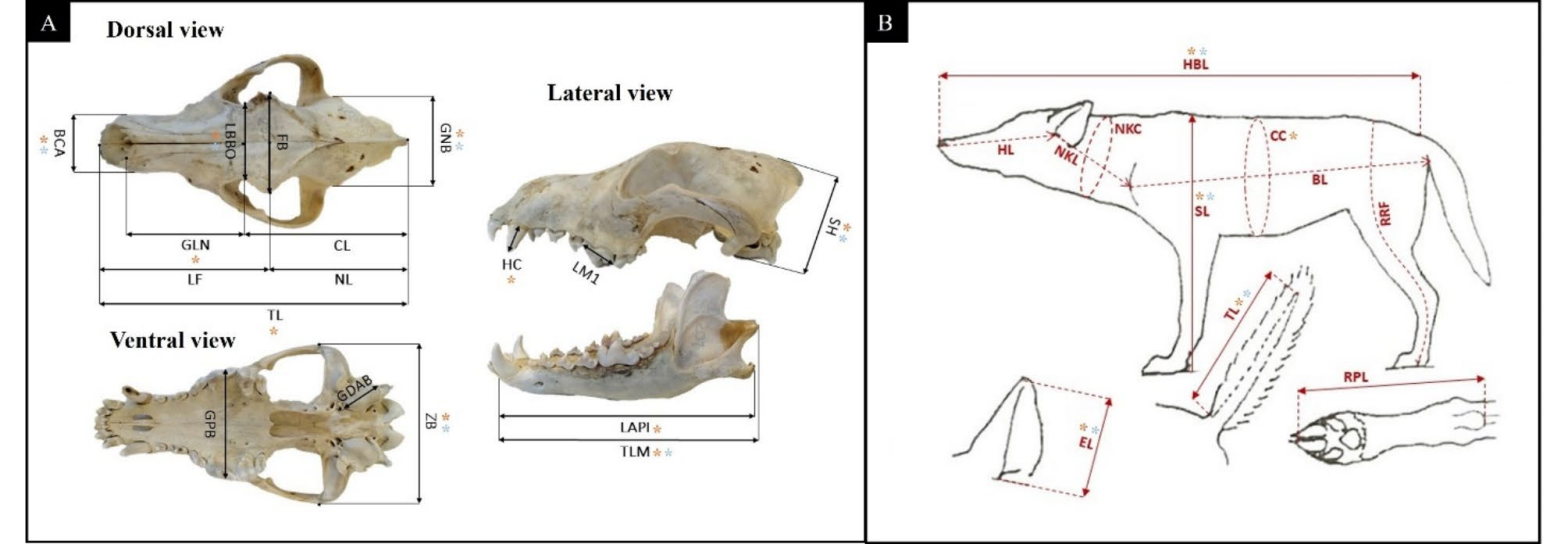
(**A**) Craniometrical parameters measured in wolf adult skulls to describe their morphometry. (**A**) Dorsal view. *TL* total length, *LF* facial length, *NL* upper neurocranium length, *GLN* maximum nasal length, *CL* cranial length, *BCA* rostrum width, *LBBO* minimum breadth between the orbits, *FB* maximum frontal breadth, *GNB* maximum neurocranium breadth. Ventral view. *GPB* greatest breadth of the palatine, *GDAB* greatest diameter of the auditory bulla, *ZB* zygomatic breadth. Lateral view. *HC* height of upper canine, *LM1* upper carnassial length, *SH* skull height, *LAPI* angular process-interdental, *TLM* total length of the mandible. (**B**) Body parameters measured in wolf adult carcasses to describe their morphology. *HBL* head and body length, *HL* head length, *NKL* neck length, *NKC* neck circumference, *SL* height at the shoulders, *CC* chest circumference, *BL* body length, *RRF* rump to rear foot pad, *EL* ear length, *TL* tail length, *RPL* rear paw length. Morphometric parameters used to compare populations in Principal Component Analyses (Figs. 5 and 6) are indicated by orange stars. Morphometric parameters used to compare among-population minimum, maximum and mean values in box plots represented in Fig. S1 and Fig. S2 are indicated by blue stars.

The 19 HW and the 8 dog (at least 24 months old) adult skulls (Table 1 and Table S1) were independently measured three times each using a 1-mm accuracy caliper (30 cm) for 17 different wolf diagnostic craniometric parameters^66,67^ (Fig. 2A and Table S2). Mean individual wolf and dog craniometric values were first compared to each other and then, using a subset of 10 shared measures (Fig. 2A and Table S2), also with those of 26 Norwegian and 44 Swedish wolves obtained from Engdal (2018)^68^, through multivariate analyses (PCA) implemented in PAST, assessing significance levels for each comparison using a multivariate test of variability (MANOVA)^65^.

Additionally, for another subset of 6 shared craniometric measures (Fig. 2A and Table S2), female and male HW average values were compared to average values of 70 Scandinavian wolves (23 females, 47 males), 186 Latvian wolves (72 females, 114 males), 78 Carpathian wolves (29 females, 49 males), 71 Polish wolves (31 females, 40 males) obtained from Engdal (2018)^68^, Andersone & Ozoliņš (2000)^69^, Okarma & Buchalczyk (1993)^70^, and comparison results were graphically visualized as box plots showing minimum, maximum and mean values plus standard deviations.

#### Museum skin morphological description

The 20 available HW skins (9 females, 11 males; Table 1 and Table S1) were visually examined to qualitatively describe the presence of two morphological traits (dark vertical bands along the back and forelimbs and the interdigital pad between the 3rd and 4th finger) typical of the Italian wolf population^32,35^, and the possible presence of other 3 phenotypical anomalies which could be interpreted as possible signals of hybridization with the domestic dog (anomalous coat colour patterns, spur on the hind legs and white claws^49,71,72^; Table S2).

#### Body measurements

The carcasses of 11 HW (6 females, 5 males) and 25 CW (8 females, 17 males) adult individuals (Table 1 and Table S1), i.e. older than 12 months when the adult size is generally reached^73^, were also morphologically examined by wildlife veterinarians during necropsy and measured for 11 specific morphometric parameters selected from those used by the Federation Cynologique International (FCI) to define breed standards^74^, as described in Fig. 2B and in Table S2. Individual HW and CW morphometric values were compared through PCA implemented in PAST, assessing comparison significance levels using a MANOVA test^65^. Similarly, for a subset of 5 shared morphometric measures (Fig. 2B and Table S2), individual HW and CW morphometric values were compared to individual values of 16 Scandinavian (7 females, 9 males) wolves obtained from Engdal (2018)^68^. Additionally, for a subset of 4 shared morphometric measures (Fig. 2B and Table S2), Italian female and male wolf average body measurement values were compared with female and male average values of 16 (7 females, 9 males) Scandinavian obtained from Engdal (2018)^68^ and 31 Eastern Serbian (12 females, 19 males), 38 Western Serbian (14 females, 24 males), 34 Bosnia-Herzegovinian (9 females, 15 males) and 103 Central Balkans (45 females, 58 males) wolves obtained from Trbojević (2016)^75^. The selection of morphometric measurements was guided by the availability of common measurements found in the literature^74^. Results were graphically visualized as box plots showing minimum, maximum and mean values plus standard deviations.

## Results

### Molecular analyses

#### Multilocus genotype reconstruction, amplification success and error rates

Following the multiple-tube protocol, after the two PCR replicates per sample per locus, all 50 HW and 56 CW tissue and blood DNA samples were successfully genotyped at all loci with less than 3 missing data, showing an average positive amplification rate ≥ 0.98 and no presence of ADO or FA. Only 1 out of 9 petrous bone DNA sample was discarded from the analyses, showing more than 80% of missing data, due to its very low concentration (0.7 ng/µl). The remaining 8 petrous bone DNA samples were successfully genotyped at all loci with less than 6 missing data, showing concentration values ranging from 1.46 to 32.8 ng/µl, and an average positive amplification rate ≥ 0.93 with ADO < 1% and no presence of FA. The authenticity of the data was upheld by the strict guidelines for ancient DNA analysis^11,76^ followed during this study and supported by the absence of DNA contamination in any of the blank extractions or negative controls included in each reaction.

Regrouping procedures indicated that these 114 successfully genotyped samples corresponded to 112 distinct 39-STR genotypes (57 males and 55 females; average positive amplification rate ≥ 0.96, ADO < 0.5% and no FA), since the genotypes of the 2 individuals that were reconstructed from 2 independent sample sources (tissues and petrous bones) perfectly matched one another at all loci (100%), supporting the authenticity of the data (Table S1).

All samples were also successfully typed at the K-locus and at the mtDNA CR, and all the detected males were successfully genotyped at the 4 Y-linked STRs (Table S1).

#### Genetic population structure and admixture analysis

**Fig. 3:**
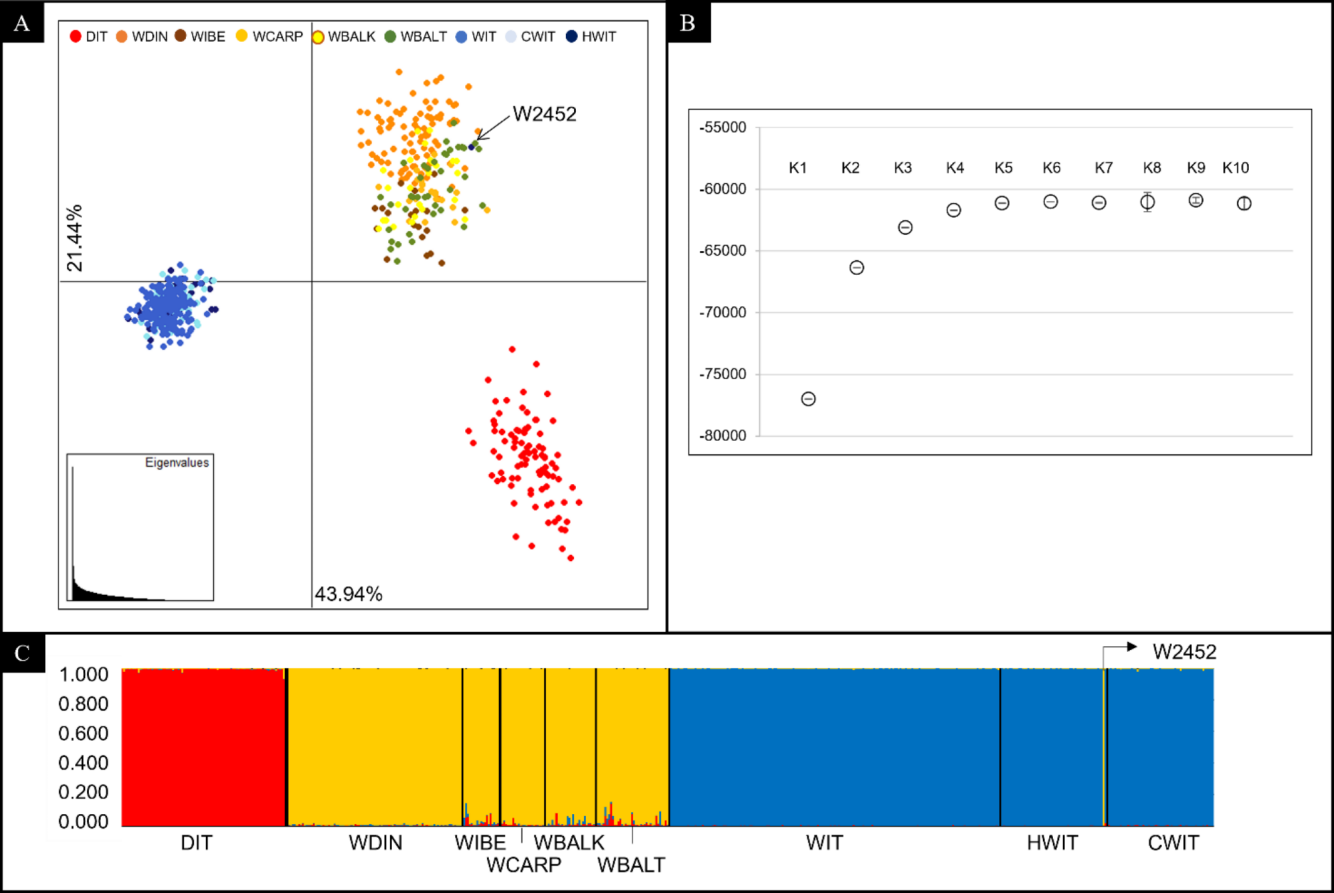
(**A**) Exploratory Principal Component Analysis (PCA) computed in Adegenet and performed using the 39-STR genotypes of 56 Historical Italian wolves HWIT (dark blue dots), 56 Contemporary Italian wolves CWIT (light blue dots), 175 reference Italian wolves (WIT, blue dots), 89 Italian dogs (DIT, red dots) and 196 European wolves from 5 geographical populations: Dinaric = WDIN, Iberian = WIBE, Carpathian = WCARP, Balkan = WBALK, Baltic = WBALT. The first component PC-I explains 43.94% of the total genetic variability and clearly separates the Italian wolf population from the European wolves and domestic dogs, while these latter two are plainly separated along the second component PC-II which explains 21.44% of the total genetic variability. (**B**) Estimated posterior probability LnP(*K*) and corresponding standard deviations of the *K* genetic clusters from 1 to 10. (**C**) Bar plotting of the individual *q*_i_-values obtained through Bayesian model-based clustering procedures implemented in Structure and performed using the 39-STR genotypes of 56 HWIT, 56 CWIT and, as reference populations, the 39-STR genotypes of 175 Italian wolves (WIT), 89 dogs (DIT) and 196 European wolves from 5 geographical populations (WDIN, WIBE, WCARP, WBALK, WBALT). Each individual is represented by a vertical line partitioned into coloured segments, whose length is proportional to the individual coefficients of membership (*q*i) to the wolf and dog clusters inferred assuming *K* = 2 clusters and using the ‘‘*Admixture*’’ and ‘‘*Independent allele frequencies*’’ models.

The preliminary multivariate analysis, performed considering the 39-STR genotypes of reference dogs, Italian and European wolves, clearly separated the three canid groups (Fig. 3A). All HW and CW 39-STR genotypes completely plotted within the reference Italian wolf cluster, with the only exception of a male HW (W2452) which grouped with the European wolves (Fig. 3A).

Multivariate analyses were strongly confirmed by the Bayesian clustering procedures implemented in Structure that showed increasing rates in the estimated posterior probability LnP(*K*) of the clusters until *K* = 3 (Fig. 3B). At *K* = 3, dogs clustered separately (*Q*_1_ = 0.994) from reference Italian (*Q*_2_ = 0.998) and the other European (*Q*_3_ = 0.980) wolves (Table S3A). HW and CW 39-STR genotypes were unambiguously assigned to the reference Italian wolf cluster (*Q*_2_ = 0.995), whereas the HW sample named W2452 confirmed to share a non-Italian origin, being its 39-STR genotype clearly assigned to the reference European wolf cluster with a *q*_i_ = 0.998 (Fig. 3C and Table S1), and showing mtDNA (W5^51^) and Y-Chr (YH15^40^) haplotypes typical of the Balkan wolf macro-population^28^ (Table S1). For these reasons, this sample was removed from subsequent admixture analyses performed considering only HW and CW, together with reference dogs and reference Italian wolves.

**Fig. 4:**
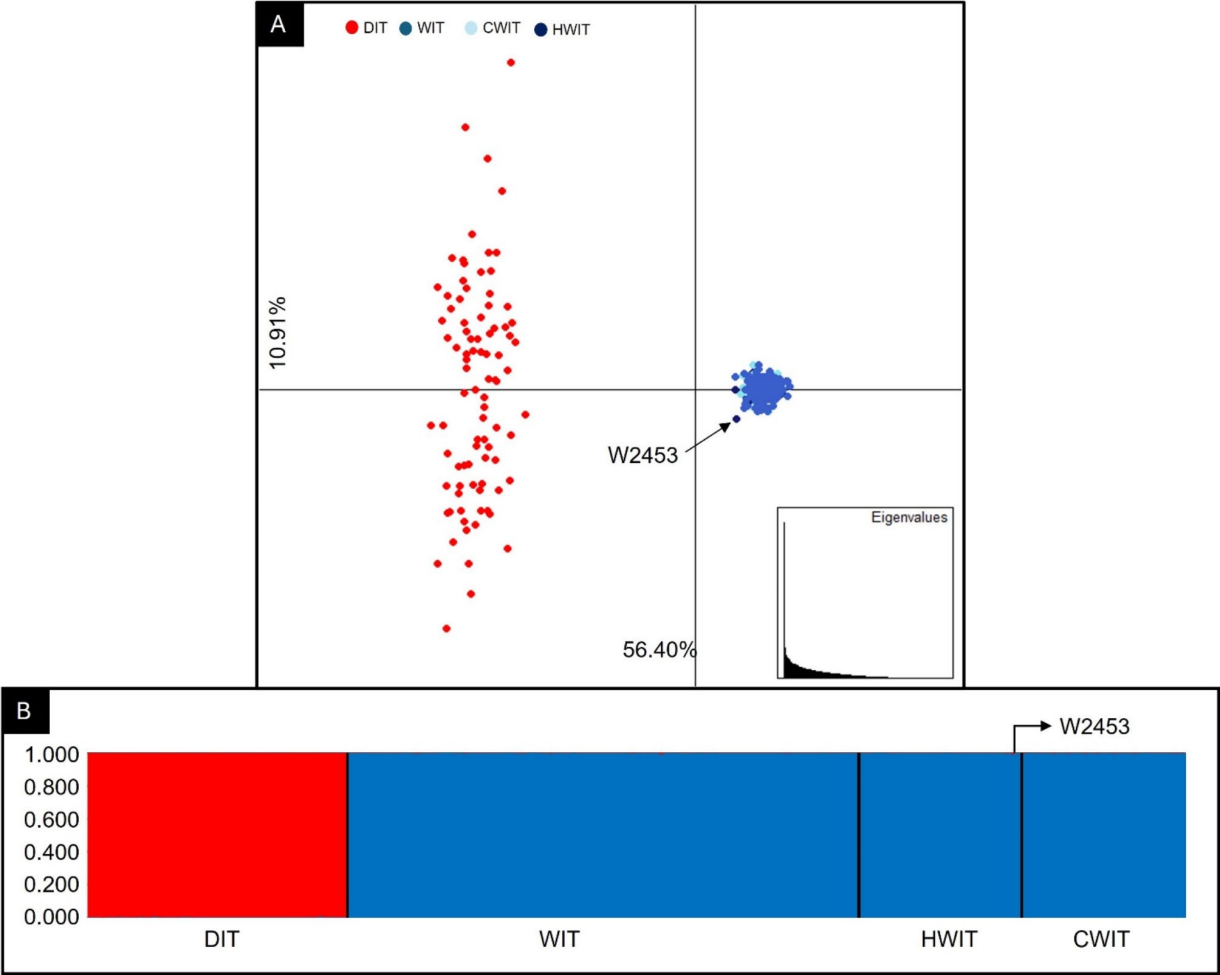
(**A**) Exploratory Principal Component Analysis (PCA) computed in Adegenet and performed using the 39-STR genotypes of 55 Historical Italian wolves HWIT (dark blue dots), 56 Contemporary Italian wolves CWIT (light blue dots), 175 reference Italian wolves (WIT, blue dots), 89 Italian dogs (DIT, red dots). The first component PC-I explains 56.40% of the total genetic variability and clearly separates the Italian wolf population from domestic dogs, while the second component PC-II which explains 10.91% of the total genetic variability, describes the genetic variability observed within these latter. (**B**) Bar plotting of the individual *q*_i_-values obtained through Bayesian model-based clustering procedures implemented in Structure and performed using the 39-STR genotypes of 55 HWIT, 56 CWIT and, as reference populations, the 39-STR genotypes of 175 Italian wolves (WIT) and 89 dogs (DIT). Each individual is represented by a vertical line partitioned into coloured segments, whose length is proportional to the individual coefficients of membership (*q*i) to the wolf and dog clusters inferred assuming *K* = 2 clusters and using the ‘‘*Admixture*’’ and ‘‘*Independent allele frequencies*’’ models.

When focusing on the Italian context, the multivariate analysis clearly separated domestic and wild reference canids with all HW and CW 39-STR genotypes completely overlapping the reference Italian wolves, with the only exception of a male HW (W2453) which plotted marginal to these latter (Fig. 4A). Bayesian clustering procedures showed that at *K* = 2 (Table S3B), reference dogs (*Q*_1_ = 0.998) clustered separately from reference Italian wolves (*Q*_2_ = 0.999). HW and CW 39-STR genotypes were unambiguously assigned to the Italian wolf cluster (*Q*_2_ = 0.999), with the exception of W2453 which was assigned to the reference Italian wolf cluster with a *q*_i_ = 0.990 (90% CI: 0.929-1.000), and thus was considered as an introgressed individual^33^ (Fig. 4B and Table S1). Additionally, the individual W2453 and the individual W0489 showed signals of dog introgression at the paternal lineage sharing a dog Y-Chr (YH06) haplotype^40^, whereas the remaining 54 males showed typical peninsular Italian wolf Y-Chr (YH17, *n* = 38, YH26, *n* = 16^40^) haplotypes^28^. None of HW and CW genotypes showed other genetic anomalies, since they all shared mtDNA CR (W14, *n* = 109, W16, *n* = 2^51^) haplotypes typical of the peninsular Italian wolf population and the absence of the 3-bp melanistic deletion at the K-locus (Table S1).

#### Genetic variability analysis

All the 39 autosomal microsatellite loci were polymorphic both in CW and HW (Table 2) with a mean number of alleles per locus of 3.85 ± 0.25 (range 2–11) in HW and 4.69 ± 0.24 (range 2–9) in CW (Table 2). Among the 195 identified alleles, 140 alleles (72%) were shared by the two groups.

**Table 2:** Genetic variability indexes estimated from the genotypes at 39 autosomal microsatellite loci of historical (HW) and contemporary (CW) Italian wolves, and, for comparative purpose, of Dinaric (WDIN), Iberian (WIBE), Carpathian (WCARP), Balkan (WBALK) and Baltic (WBALT) wolves (n = sample size).

Group (*n*)	H_O_	H_E_	*N*_A_	*N*_E_	*N*_AR_	*N*_*R*_	*N*_*P*_	*N*_DHW_	F_IS_
HW (55)	0.47 (0.04)	0.49 (0.04)	3.85 (0.25)	2.34 (0.15)	3.18 (0.18)	42	3	3	0.064***
CW (56)	0.48 (0.03)	0.51 (0.03)	4.69 (0.24)	2.39 (0.15)	3.63 (0.16)	65	6	7	0.067***
WDIN (92)	0.64 (0.02)	0.70 (0.02)	6.69 (0.28)	3.69 (0.22)	5.07 (0.20)	92	15	6	0.087***
WIBE (19)	0.53 (0.04)	0.61 (0.03)	4.54 (0.25)	3.09 (0.19)	4.32 (0.24)	20	7	1	0.166***
WCARP (23)	0.61 (0.03)	0.62 (0.02)	4.64 (0.20)	2.94 (0.14)	4.24 (0.17)	44	2	7	0.035*
WBALK (26)	0.65 (0.02)	0.71 (0.02)	6.56 (0.31)	3.91 (0.21)	5.85 (0.25)	62	14	4	0.110***
WBALT (38)	0.67 (0.02)	0.74 (0.01)	7.28 (0.27)	4.22 (0.20)	6.00 (0.20)	91	21	7	0.104***

H_O_: observed heterozygosity, H_E_: expected heterozygosity, N_A_: number of alleles, N_E_: number of effective alleles, N_AR_: allelic richness based on 19 individuals, N_R_: number of rare alleles with frequencies 0.001 < freq. < 0.05, N_P_: number of private alleles per population, N_HWD_: number of loci in Hardy-Weinberg disequilibrium (with a *p* < 0.001 after Bonferroni correction); standard errors are in parentheses. Departures from Hardy-Weinberg equilibrium were assessed for each population from the average multilocus *F*_IS_ values (the average individual inbreeding coefficient within each population) computed after 10,000 random permutations using Genetix (***P < 0.001; **P < 0.01; *P < 0.05).

Mean HW H_O_ (0.47 ± 0.04), H_E_ (0.49 ± 0.04), Ne (2.34 ± 0.15) and CW H_O_ (0.48 ± 0.03), H_E_ (0.51 ± 0.03), N_E_ (2.39 ± 0.15) values were not significantly (p-values > 0.05; *t*-tests) different (Table 2).

Similarly, mean HW N_A_ (3.85 ± 0.25) and mean HW N_AR_ (3.18 ± 0.18) values were not significantly different (p-value = > 0.05; *t*-tests) from mean CW N_A_ (4.69 ± 0.24) and mean CW N_AR_ (3.63 ± 0.16) values (Table 2).

Departures from HWE were detected for 3 loci in HW and for 7 loci in CW, due to significant differences between expected and observed heterozygotes.

When compared to the other analyzed European wolf populations, HW/CW showed always lower H_o_, N_A_, N_AR_ values, with significant (p-values < 0.05) differences with WDIN, WBALK, WBALT (Table S4).

All the analyzed European wolf populations, as well as HW and CW, showed possible signals of inbreeding as indicated by significant positive *F*_IS_ values due to significant heterozygote deficits (Table 2).

### Morphometric analyses

#### Skull morphometry

**Fig. 5:**
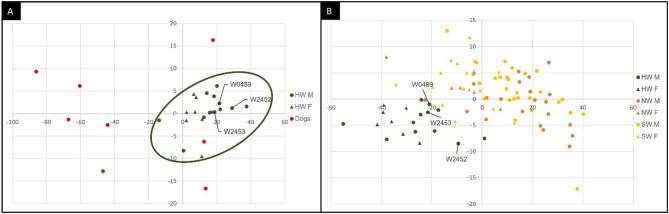
Principal Component Analysis (PCA) computed in PAST using: (**A**) 17 craniometrical parameters (see Fig. 2A for details) measured to describe the morphometry in adult skulls of 12 Historical Italian male wolves (HW M, green dots), 8 Historical Italian female wolves (HW F, green triangles) and 8 domestic dogs (red dots); (**B**) 10 craniometrical parameters (signed with yellow asterisk in Fig. 2A) measured to describe the morphometry in adult skulls of 12 Historical Italian male wolves (HW M, green dots), 8 Historical Italian female wolves (HW F, green triangles), 20 Norwegian male wolves (NW M, orange dots), 6 Norwegian female wolves (NW F, orange triangles), 27 Swedish male wolves (SW M, yellow dots), 17 Swedish female wolves (SW F, yellow triangles). The Balkan wolf (W2452) and the 2 dog-introgressed (W0489 and W2453) HW samples are labelled with their individual codes.

The first morphometric PCA, performed using 17 diagnostic craniometric measures, showed that the 20 HW, which provided reliable multilocus genotypes (Table S1), and the 8 dogs were significantly separated (*P*_MANOVA_< 0.001), with only 1 dog plotting close to wolves. The Balkan and the 2 dog-introgressed HW individuals completely fell into the wolf distribution (Fig. 5A).

The second morphometric PCA, performed using only 10 shared diagnostic craniometric measures (Table S2), showed that the HW (in the left bottom part of the graph) resulted significantly separated (*P*_MANOVA_< 0.0001) also from the 70 Scandinavian wolves (in the right upper part of the graph), confirming their average smaller skull sizes both considering (20 HW) and excluding (17 HW) the Balkan and the 2 dog-introgressed HW individuals which plotted marginal to the HW distribution (Fig. 5B).

Additionally, when considering a subset of 6 shared craniometric measures (Fig. 2A and Table S2), HW male and female average values and their standard deviations confirmed to be smaller than Scandinavian and Latvian males and females in all comparisons (Fig. S1) and also smaller than Polish and Carpathian males and females for more than half measures (Fig. S1).

#### Museum skin morphological description

The morphological qualitative description of the 19 available HW skins (Table 1 and Table S1), which provided reliable multilocus genotypes, showed that the dark vertical bands along the back and forelimbs, typical of the peninsular Italian wolf population^77^, were present in all the examined individuals, the interdigital pad was observed in only 2 (12.5%) samples, whereas the spur and white claws were never detected. As expected, the Balkan HW showed an extended black spot on the tail, typical of the European wolf populations but unusual in the peninsular Italian wolves.

#### Body measurements

**Fig. 6:**
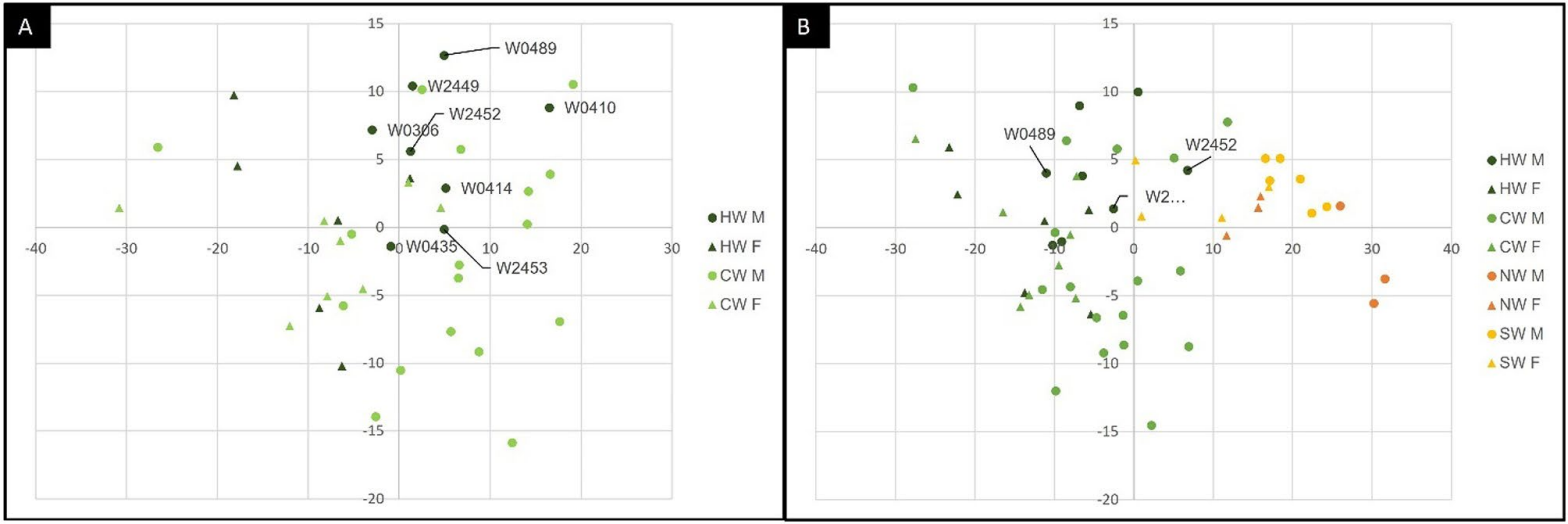
Principal Component Analysis (PCA) computed in PAST using: (**A**) 11 morphometric parameters (see Fig. 2B for details) measured to describe the morphology in adult carcasses of 8 Historical Italian male wolves (HW M, dark green dots), 6 Historical Italian female wolves (HW F, dark green triangles), 17 Contemporary Italian male wolves (CW M, light green dots), 8 Contemporary Italian female wolves (CW F, light green triangles); (**B**) 5 morphometric parameters (signed with * in Fig. 2B) measured to describe the morphology in adult carcasses of 8 Historical Italian male wolves (HW M, dark green dots), 6 Historical Italian female wolves (HW F, dark green triangles), 17 Contemporary Italian male wolves (CW M, light green dots), 8 Contemporary Italian female wolves (CW F, light green triangles), 3 Norwegian male wolves (NW M, orange dots), 3 Norwegian female wolves (NW F, orange triangles), 6 Swedish male wolves (SW M, yellow dots), 4 Swedish female wolves (SW F, yellow triangles). The Balkan wolf (W2452) and 2 dog-introgressed (W0489 and W2453) HW samples are labelled with their individual codes.

A first body measure PCA, performed using 11 diagnostic morphometric parameters measured during necropsy, showed that the 14 HW, which provided reliable multilocus genotypes, did not significantly differ (*P*_MANOVA_= 0.966) from the 25 CW, with the former group completely overlapping the latter (Fig. 6A).

Conversely, a second body size PCA, performed using 5 among-population shared morphometric parameters, confirmed that overall, the total 39 peninsular Italian wolves (in the left part of the graph) significantly differed (*P*_MANOVA_< 0.00001) from the 16 available Scandinavian (10 Norwegian and 6 Swedish) wolves (in the right part of the graph), both considering (for a total of 14 HW) and excluding (for a total of 11 HW) the Balkan and the 2 dog-introgressed HW individuals which plotted marginal to the HW distribution, with only a partial slight overlap between a few Scandinavian females and a few peninsular Italian males (Fig. 6B).

Finally, when considering a subset of 4 shared body measures (Fig. 2B and Fig. S2), peninsular Italian (HW plus CW) male and female average values and their standard deviations were confirmed to be smaller than average values and standard deviations available for Scandinavian, central-Balkan and Dinaric males and females in most comparisons (Fig. S2).

## Discussion

Thanks to a multidisciplinary approach based on the availability of a well-preserved museum historical collection of peninsular Italian wolf skins and skulls, emerging ancient DNA extraction techniques^41^ and a highly diagnostic genetic multi-marker panel^33^, for the first time, we genetically and morphologically described the most divergent wolf population in Europe, the peninsular Italian wolf population^28^. Furthermore, we investigated whether its genetic variability has significantly changed from 1990s until nowadays, trying to overcome or, at least, minimize, the intrinsic numerical and qualitative challenges linked to the analyses of historical samples^78^.

### Molecular analyses

Individual multilocus genotypes were reconstructed by analyzing fresh DNA obtained from carcasses and blood samples, using commercial silica-based extraction methods, and from the petrous bone of museum specimens, using recently in-house optimized extraction methods^44^, modified from Dabney et al. (2013)^43^. The applied commercial silica-based DNA extraction method and the multiple-tube protocol allowed us to obtain very high amplification success rates and neither ADO nor FA errors for both historical and modern tissue and blood samples, confirming very powerful genotyping performances of well-preserved biological materials, even when collected almost 30 years ago^79^. The applied in-house optimized ancient material extraction method and the strict guidelines for ancient DNA analyses allowed us to obtain reliable individual multilocus genotypes with negligible error rates and very high amplification and genotyping success rates, notably higher than those usually obtained from non-invasively collected materials^52,63^ and comparable to those obtained from fresh muscular tissues^28,40^, even for about 90% of the petrous bone DNA samples. These results suggest the opportunity for a successful use of this sample type in future population dynamic monitoring projects based on the analysis of canid DNA contained in very degraded wild carcasses and museum historical samples. Additionally, this sample type could be used in paleogenomic studies planned to better investigate the evolutionary histories and demographic trajectories of taxa through time^12,80^.

Both multivariate and Bayesian assignment procedures, performed using the obtained HW and CW multilocus genotypes together with the genotypes of reference domestic dogs, European and Italian wolves, showed no substructure between HW and CW, which completely overlap with the reference Italian wolf population, resulting clearly separated from both European wolves and dogs, with the only exception of 1 HW sample. This sample was unquestionably assigned to the European wolf cluster and showed mtDNA and Y-Chr typical of the Balkan wolf macro-population^28^, probably indicating a captive-bred individual escaped from a wildlife recovery center located near its sampling location.

All the remaining analyzed HW and CW did not show any evident traces neither of non-Italian genome nor of recent hybridization with the dogs, with only 2 HW individuals sharing slight signals of domestic introgression more ancient than the third-fourth backcrossing generations^33^.

These findings clearly confirm, despite representing only a moderately resolved snapshot of the non-coding variability within the *Canis* genome, the high diagnostic power of the applied multilocus bi and uniparental marker panel for both individual and taxon identification. Therefore, such panel could be successfully used not only for conservation purposes, detecting the presence of potential anthropogenic wolf-dog admixed individuals, but also for forensic applications, recognizing animals dispersing from other populations, as well as animals escaped from zoos or wildlife recovery centers^33,46^.

Our results about the standing genetic variation showed that most of the detected alleles were shared between HW and CW, and that all the variability indexes were not significantly different between the 2 groups due to random drift, as expected since we analyzed samples from the historical core distribution area (central Apennines) of the species, where it never disappeared, remaining isolated even during the re-expansion phase without migrants from other populations^81^. Additionally, our variability estimates are consistent with outcomes from other molecular studies about the Italian wolf population origin and dynamics, based on the same type and number of markers^28,40^.

Only HW mean observed allele numbers and HW mean allele richness values from this study were lower than those observed in CW, likely due to a major number of private alleles observed in the latter. These private alleles might have not been previously detected in HW because they were present at very low frequencies, and detected in CW only later, as the result of their spreading by dispersers and floaters. However, mean effective numbers of alleles were almost identical in the two sample groups, confirming that, despite the numerical demographic increase observed during the last decades^27,86^, the peninsular Italian wolf population continues to show low genetic variability. These findings suggest that its long-lasting isolation in peninsular Italy, started during the last glacial maximum^28,38^, and likely exacerbated during the recent anthropogenic bottleneck of the last century (early 1900s), left not negligible genetic signatures due to the consequent inbreeding and genetic drift at the analyzed neutral loci^30^ as shown by the significant heterozygote deficit (positive *F*_IS_) observed in the two sample groups. Our results would seem to corroborate preliminary genomic analyses performed on a few individuals collected in the same area of this study, which showed high signatures of inbreeding, and a non-negligible genetic load^87^. The slightly higher number of observed alleles in the CW might be linked to a random subsampling of HW or be the legacy of some rare alleles remaining in the source population whose frequencies gradually increased in the re-expanding inbred population after the bottleneck. These findings clearly suggest the need to continuously monitor the population dynamics, even using genomic data, to better comprehend its variability patterns through time. However, results from our study should be taken with caution since they provide a comprehensive overview of only the post-20th century bottleneck of the peninsular Italian wolf population. Unfortunately, we could not include any Holocene pre-bottleneck specimens, as reported in other studies on similarly inbred populations^85^, due to the very limited availability of a representative sample of the post-glacial period population^39^.

When compared to the other analyzed wolf populations, the peninsular Italian wolf confirmed to be one of the less genetically variable^28,88,89^. Indeed, Italian samples showed always a lower genetic variability, with significant differences with WDIN, WBALK, WBALT, but not with WIBE, which suffered a similar severe anthropic bottleneck^85^, and WCARP, probably because of the restricted number of Carpathian wolves we analyzed.

### Morphometric analyses

All the analyzed craniometric measures revealed to be highly performing in (a) discriminating wolves and dogs, with the only exception of 1 German shepherd dog which plotted close to wolves, (b) as well as in distinguishing the peninsular historical Italian wolf population from all the other European wolf populations, including those deeply inbred such as the Scandinavian population^83^. Additionally, craniometric data confirm previous findings^35^ reporting a marked sex dimorphism and an average smaller skull size of the peninsular Italian wolf population compared to most of the other European wolf populations, highly consistent with the Bergmann’s rule^89^, according to which widely distributed species can show larger size in colder environmental contexts. Moreover, the body size observed in peninsular Italian wolves might also reflect local environmental adaptations resulting more ecologically advantageous in Mediterranean forested areas and might ensure more chances to survive to anthropic pressures in highly human-dominated landscapes. Unfortunately, it was not possible to evaluate any skull morphological differences between HW and CW because of the unavailability of skulls from the contemporary carcasses we analyzed.

Marked significant differences between peninsular Italian and European wolves emerged also from the comparative analyses performed using body measures. However, when considering the peninsular Italian population alone, body measures strictly confirmed outcomes derived from genetic analyses since they did not significantly differ between HW and CW, suggesting neither selective ecological pressures nor random morphological mutations fixed during the most recent anthropogenic bottleneck.

Finally, all the HW pelage patterns, extrapolated from the examined available skins, including the one belonging to the detected non-Italian individual, showed the typical phenotypical features of the population of origin and no evident morphological anomalies possibly linked to wolf-dog hybridization such as spurs and white claws^71^.

To overcome the caveats emerging from the morphological analyses performed in the current study mainly due to the limited availability and comparability of datasets and better describe the phenotypical variability of the species, it would be useful to encourage the application of standardized diagnostic protocols based on informative morphological measure sets, shared among local wildlife management authorities and research institutions^34^.

However, all these findings clearly confirm that the peninsular Italian wolf population represents a worldwide uniqueness from both the genetic and morphological point of views^28,35^, showing a reduced genetic variability and reduced body sizes when compared to other wolf populations, the former caused by the long-lasting geographic isolation the population suffered, and the latter mainly linked to ecological and environmental factors.

## Conclusions

Our multidisciplinary approach clearly demonstrates the ever-increasing importance of well-preserved historical museum collections. When appropriately genetically and morphologically analyzed and compared to contemporary samples, these collections can significantly contribute to clarifying both historical and actual population dynamics of threatened taxa, allowing us to better plan the most appropriate conservation measures.

Additionally, our multidisciplinary approach, exploiting the recently developed paleogenomic techniques^21^, which make available and comparable an increasing number of not only modern and historical but also ancient entire mitogenomes and whole nuclear genomes^21^, could further contribute to definitively explain the origin and the evolutionary patterns of the genetic and morphological variability of the most worldwide divergent and peculiar wolf population, the Italian subspecies, which continues to fascinate conservation biologists for its resilience to both natural and anthropic threats^34,90^.

## Electronic supplementary material

Below is the link to the electronic supplementary material.

Supplementary Material 1

Supplementary Material 2

## Data Availability

The majority of the data generated and analyzed during the current study are presented within the article or in Supplementary information files. The raw data are available from the corresponding author on reasonable request.
