# Supplementary material for: Museomics and morphological analyses of historical and contemporary peninsular Italian wolf (Canis lupus italicus) samples
Source: Sci Rep. 2025 Feb 4;15:4232. doi: 10.1038/s41598-024-84319-x (PMC11794570; doi:10.1038/s41598-024-84319-x)
Supplement: Supplementary file 2 — Supplementary Material 2 [file 41598_2024_84319_MOESM2_ESM.pdf]

Museomics and morphological analyses of historical and contemporary peninsular Italian wolf (*Canis lupus italicus*) samples

Supplementary information

Supplementary Table S1 – Field information (sample identification, sample type, sampling location in the Italian Regions, sampling year, individual age, skull, skin and body measure availability) and molecular identifications (taxon, sex, results from the Bayesian model-based assignment tests performed in STRUCTURE, mtDNA and Y-STR haplotypes) of historical (HW) and contemporary (CW) samples analysed in the study. ID-S: sample identification; mtDNA-H: control region of mitochondrial DNA haplotype identification as named in Randi et al. (2000); YH: Y-linked STR haplotype identification as named in Randi et al. (2014); K-locus melanistic deletion at the *β-defensin CBD103* gene: K^+/K^+ homozygote wild-type (no deletion), K^+/K^B heterozygote for the melanistic deletion; q_{WIT}: individual q_i assignment value to the Italian wolf group and corresponding 90% confidence interval (CI) obtained by STRUCTURE assuming $K = 2$ or $K = 3$ genetic clusters and using the "*Admixture*" and "*Independent allele frequencies*" models. ND: not detected; NA: not available; M: male; F: female.

Supplementary Table S2 – Craniometric, morphometric and morphological traits measured or observed, respectively, in adult skulls from historical Italian wolves available at the ISPRA natural history museum, adult carcasses examined during veterinary necropsy of both historical and contemporary wolves, and skins obtained from historical Italian wolves available at the ISPRA natural history museum. Type of analyses performed with the different craniometric and morphometric traits, parameter acronyms and their description are reported.

Supplementary Table S3 – Average membership proportions Q_i in their original sampling group (in bold) with 90% confidence intervals (CI) for the nine analysed canid groups, estimated from the model-based Bayesian assignment analyses performed in STRUCTURE, assuming K=3 genetic clusters (A) or $K = 2$ genetic clusters (B) and using the "*Admixture*" and "*Independent allele frequencies*" models as parameter settings. Data comprise the 39-STR autosomal genotypes of historical (HWIT) and contemporary (CWIT) Italian wolves together with domestic Italian dogs (DIT) and (A) six (Italian (WIT), Dinaric (WDIN), Iberian (WIBE), Carpathian (WCARP), Balkan (WBALK), Baltic (WBALT) wolf populations or (B) only the Italian wolf population. Average Q_i were obtained concatenating the data from the four independent runs using CLUMPAK.

Supplementary Table S4 – Results from the Mann-Whitney pairwise comparisons of genetic variability indexes among the seven analyzed canid groups, performed through the ANOVA analyses. H_O: observed heterozygosity; N_A: number of alleles; N_E: number of effective alleles; N_{AR}: allelic richness. Comparisons were performed on the 39-STR autosomal genotypes of historical (HWIT) and contemporary (CWIT) Italian wolves, Dinaric (WDIN), Iberian (WIBE), Carpathian (WCARP), Balkan (WBALK) and Baltic (WBALT) wolf populations. Significative p-values are in bold.

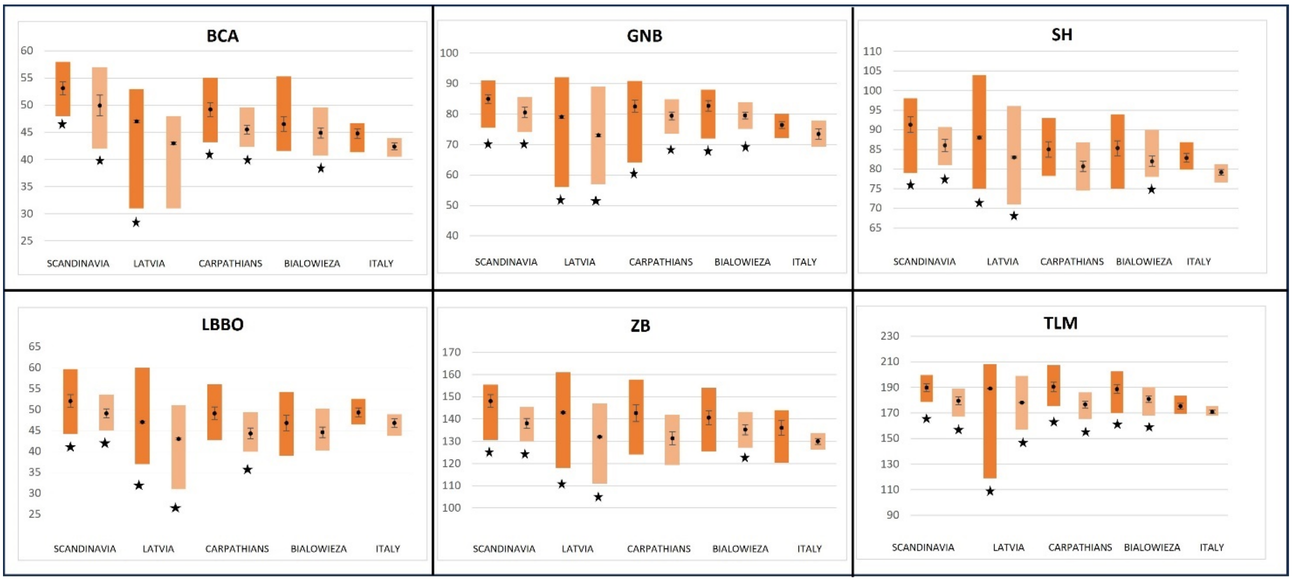

Supplementary Figure S1. Box plots showing minimum, maximum and mean values, plus standard deviations, of six craniometric parameters (signed with * in Fig. 1A) measured to describe the morphometry in adult skulls of 17 Italian wolves (nine males and eight females), 70 Scandinavian wolves (47 males and 23 females), 186 Latvian wolves (114 males and 72 females), 78 Carpathian wolves (49 males and 29 females), 71 Polish wolves (40 males and 31 females). Male and female wolves are represented, in each population, respectively, in dark and light orange. Stars indicate population measures whose standard deviation limits do not overlap those of the Italian wolf population.

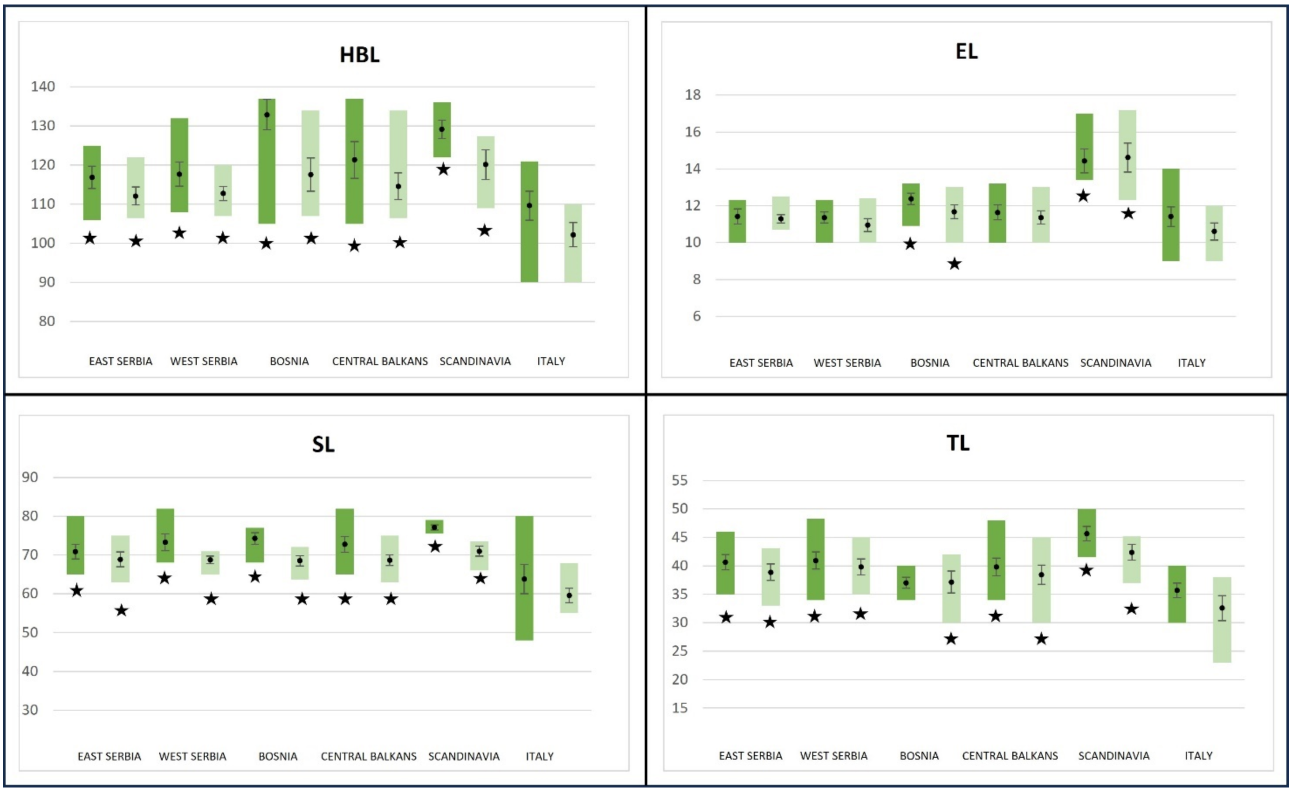

Supplementary Figure S2. Box plots showing minimum, maximum and mean values, plus standard deviations, of four morphometric parameters (signed with * in Fig. 1B) measured to describe the morphology in adult carcasses of 36 Italian wolves (22 males and 14 females), 31 Eastern Serbian (19 males and 12 females), 38 Western Serbian (24 males and 14 females), 34 Bosnia-Herzegovinian (15 males and nine females), 103 Central Balkans (58 males and 45 females) and 16 Scandinavian (nine males and seven females) wolves. Male and female wolves are represented, in each population, respectively, in dark and light green. Stars indicate population measures whose standard deviation limits do not overlap those of the Italian wolf population.

References

Andersone, Ž. & Ozoliņš, J. Craniometrical characteristics and dental anomalies in wolves *Canis lupus* from Latvia. *Acta Theriol*. **45**, 549–558 (2000).

Caniglia, R. *et al*. A standardized approach to empirically define reliable assignment thresholds and appropriate management categories in deeply introgressed populations. *Sci. Rep.* **10**, 2862 (2020).

Engdal, V. A. Phenotypic variation in past and present Scandinavian wolves (*Canis lupus L*.). Master Thesis in Ecology and evolution. Faculty of Mathematics and Natural Sciences, University of Oslo (2018).

Okarma, H. & Buchalczyk, T. Craniometrical characteristics of wolves *Canis lupus* from Poland. *Acta Theriol*. **38**, 253–262 (1993).

Trbojević, I. Sexual dimorphism and population differentiation of the wolf (*Canis lupus*) based on morphometry in the Central Balkans. *North-West. J. Zool.* **12**, 349-355. http://biozoojournals.ro/nwjz/index.html (2016).
